# Comparison of Cryoballoon vs. Pulsed Field Ablation in Patients with Symptomatic Paroxysmal Atrial Fibrillation (SINGLE SHOT CHAMPION): Study protocol for a randomized controlled trial

**DOI:** 10.1016/j.hroo.2024.05.008

**Published:** 2024-06-03

**Authors:** Jens Maurhofer, Thomas Kueffer, Sven Knecht, Gregor Thalmann, Patrick Badertscher, Nikola Kozhuharov, Philipp Krisai, Corinne Jufer, Salik ur Rehman Iqbal, Dik Heg, Helge Servatius, Hildegard Tanner, Michael Kühne, Laurent Roten, Christian Sticherling, Tobias Reichlin

**Affiliations:** ∗ Department of Cardiology, Inselspital – University Hospital Bern, University of Bern, Bern, Switzerland; † Department of Cardiology, University Hospital Basel, University of Basel, Basel, Switzerland; ‡ Department of Clinical Research, University of Bern, Bern, Switzerland

**Keywords:** Atrial fibrillation, Pulmonary vein isolation, Pulsed field ablation, Cryoballoon ablation, Implantable cardiac monitoring

## Abstract

**Background:**

Single-shot devices are increasingly used for pulmonary vein isolation (PVI) in atrial fibrillation (AF). The Arctic Front cryoballoon is the most frequently used single-shot technology. A recently developed novel pulsed field ablation (PFA) device (FARAPULSE) has been introduced with the aim to improve procedural safety and efficacy.

**Objective:**

This study will compare the novel FARAPULSE PFA device and the Arctic Front cryoballoon for first PVI in patients with symptomatic paroxysmal AF.

**Methods:**

SINGLE SHOT CHAMPION is a multicenter, randomized controlled trial with blinded endpoint adjudication by an independent clinical events committee. Overall, 210 patients with paroxysmal AF undergoing their PVI are randomized 1:1 between PFA and cryoballoon ablation. Continuous rhythm monitoring with an implantable cardiac monitor is performed in all patients.

**Results:**

The primary endpoint is time to first recurrence of any atrial tachyarrhythmia (AF and/or organized atrial tachyarrhythmia) lasting ≥120 seconds and identified by the implantable cardiac monitor within 91 and 365 days postablation. The composite procedural safety endpoint includes cardiac tamponade requiring drainage, persistent phrenic nerve palsy, vascular complications requiring intervention, stroke/transient ischemic attack, atrioesophageal fistula, and death occurring during or up to 30 days after the procedure. Key secondary endpoints include (1) increase in high-sensitivity troponin on day 1 postablation, (2) analysis of postablation 3-dimensional electroanatomic mapping (first 25 patients per study group), (3) AF burden, and (4) quality-of-life changes.

**Conclusion:**

SINGLE SHOT CHAMPION will evaluate the efficacy and safety of PVI using the novel FARAPULSE PFA for patients with symptomatic paroxysmal AF.

## Graphical abstract

**Figure:**
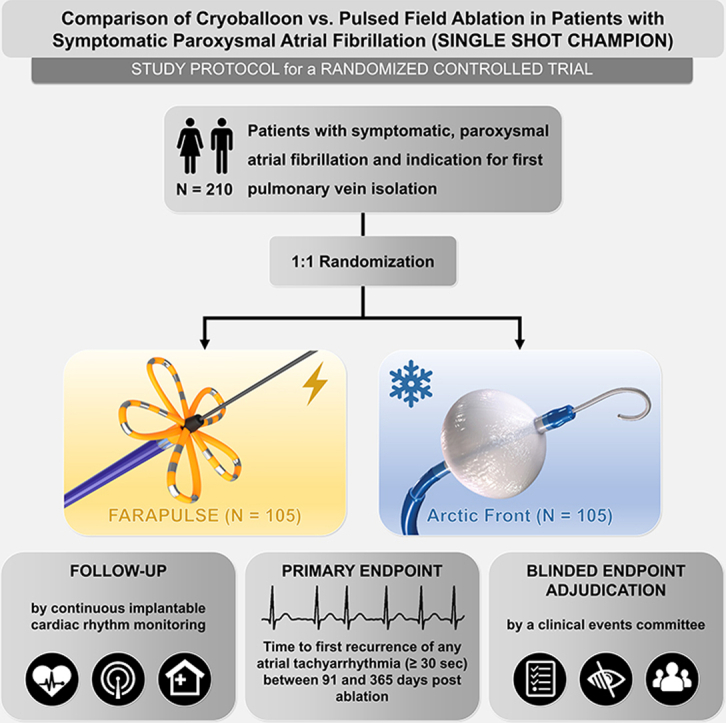
Comparison of Cryoballoon vs. Pulsed Field Ablation in Patients with Symptomatic Paroxysmal Atrial Fibrillation (SINGLE SHOT CHAMPION) – study protocol for a randomized controlled trial

**Key Findings**

▪ SINGLE SHOT CHAMPION is a multicenter, randomized controlled trial with blinded endpoint adjudication by an independent clinical events committee.
▪ The novel FARAPULSE pulsed field ablation device for pulmonary vein isolation has been introduced with the aim to improve procedural safety and efficacy.
▪ SINGLE SHOT CHAMPION will compare the novel FARAPULSE pulsed field ablation device and the well-established Arctic Front cryoballoon for first pulmonary vein isolation in patients with symptomatic paroxysmal atrial fibrillation.
▪ The primary efficacy endpoint is time to first recurrence of any atrial tachyarrhythmia detected on an implantable cardiac monitor.

## Introduction

Pulmonary vein isolation (PVI) with cryoballoon ablation is a well-established treatment for symptomatic atrial fibrillation (AF) and is superior to antiarrhythmic drug therapy in maintaining sinus rhythm and relief of symptoms related to AF.^1^, ^2^, ^3^, ^4^, ^5^ Cryothermal ablation for PVI freezes tissue to create lesions in the myocardium, with the aim to electrically isolate the pulmonary veins (PVs). Despite the ever-expanding landscape of arrhythmia mapping and catheter ablation technologies and improved imaging modalities to characterize the arrhythmia substrate, recurrence of AF after the ablation remains common and is mainly driven by PV reconnections.^6^, ^7^, ^8^ This indicates the need for an ablation modality that leads to lesions of higher durability. Additionally, there are potential risks and complications associated with thermal energy ablation affecting adjacent tissues (eg, esophagus, phrenic nerve).^9^

Currently, the Arctic Front cryoballoon (Medtronic) is the most frequently used single-shot technology for PVI.^10^ Hence, it is the benchmark for upcoming single-shot ablation technologies. A novel single-shot ablation technology has recently been introduced (FARAPULSE; Boston Scientific) with the aim to address safety and efficacy limitations of thermal ablation.^11^^,^^12^ Unlike thermal ablation techniques such as cryoballoon ablation, pulsed field ablation (PFA) utilizes high-voltage electrical fields to induce irreversible electroporation and cellular necrosis in target tissues. The unique characteristic of PFA lies in its ability to achieve selective myocardial tissue ablation while minimizing damage to adjacent structures such as the esophagus and phrenic nerve.^13^, ^14^, ^15^

Initial clinical experiences with the FARAPULSE PFA catheter have suggested comparable safety and efficacy of PVI in patients with paroxysmal AF.^16^, ^17^, ^18^, ^19^, ^20^ However, many of these studies were constrained by retrospective designs, absence of randomization, and/or fragmentary rhythm monitoring protocols during posttreatment surveillance. In cases of intermittent arrhythmias, the absence of continuous rhythm monitoring (ICM) throughout follow-up leads to overestimation of arrhythmia-free survival rates and misclassification errors, thereby potentially compromising the precision of comparative risk assessments.^21^, ^22^, ^23^, ^24^

The SINGLE SHOT CHAMPION study is a prospective multicenter, randomized controlled trial with blinded ICM-based endpoint adjudication and will compare the efficacy and safety of the novel FARAPULSE PFA device and the well-established Arctic Front cryoballoon in patients with symptomatic paroxysmal AF undergoing their first PVI.

## Methods

### Study design

**Figure 1:**
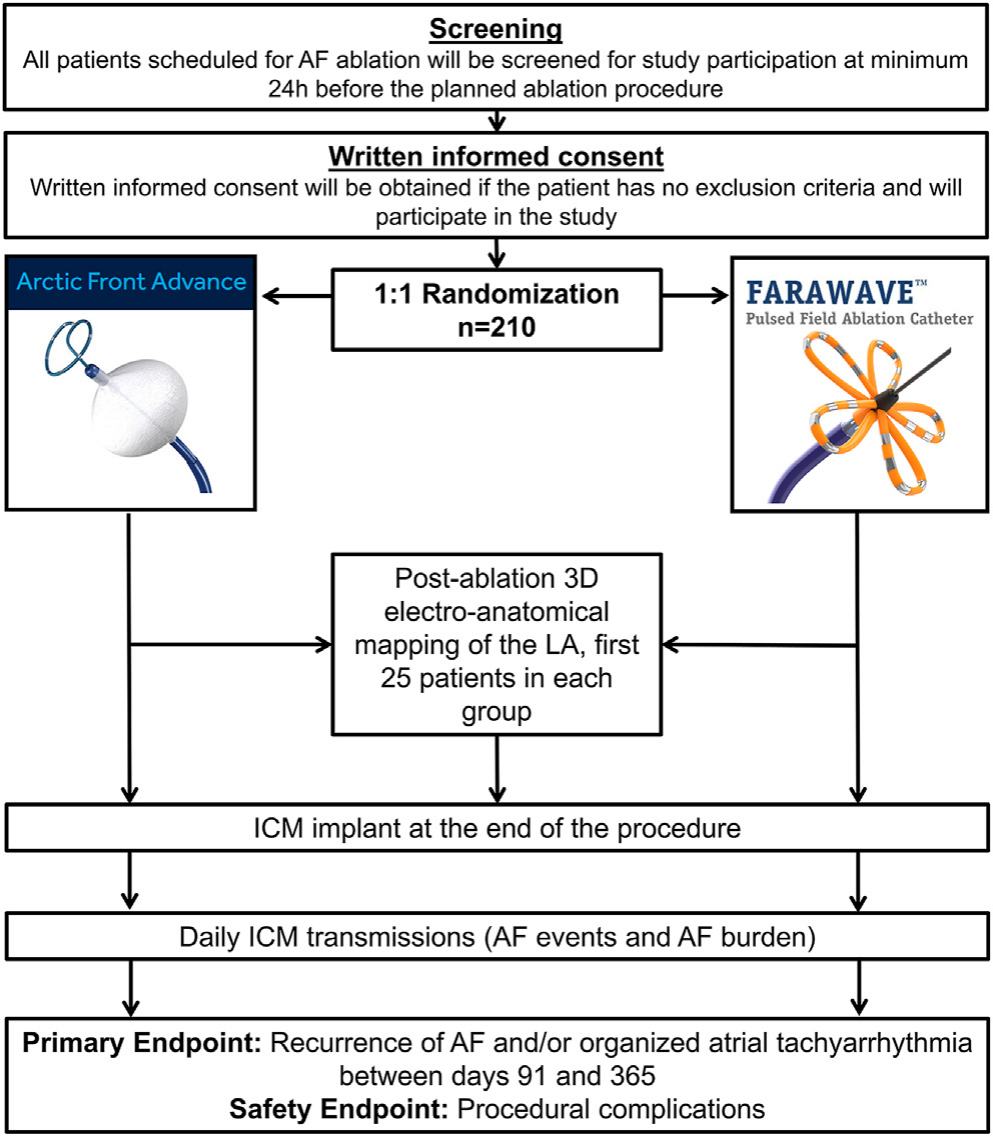
Flowchart of clinical trial design. 3D = 3-dimensional; AF = atrial fibrillation; EP = endpoint; LA = left atrial; ICM = implantable cardiac monitor.

SINGLE SHOT CHAMPION (NCT05534581) is an investigator-initiated, randomized controlled (1:1), parallel, noninferiority, prospective, open-label phase 4 trial with blinded endpoint adjudication performed in 2 clinical centers in Switzerland. The objective of the SINGLE SHOT CHAMPION study is to assess the effectiveness and safety of the FARAPULSE PFA catheter compared with the Arctic Front cryoballoon for initial PVI procedure in patients with symptomatic paroxysmal AF. Considering that cryoballoon ablation is the established standard for PVI with single-shot ablation while the FARAPULSE PFA catheter represents a novel technology, this trial adopts a noninferiority design. The study flowchart is given in Figure 1.

### Study population

Patients 18 years of age and older, diagnosed with paroxysmal AF confirmed by a 12-lead electrocardiogram or Holter monitor (lasting ≥30 seconds) within the previous 24 months, and suitable for PVI in accordance with current AF guidelines, undergo screening and are included if no exclusion criteria are met and written informed consent is provided.^2^ Paroxysmal AF is defined as per current guidelines, indicating AF that spontaneously converts to sinus rhythm within 7 days or via pharmacological or electrical cardioversion.^2^ Key exclusion criteria encompass persistent AF, prior left atrial (LA) ablation or surgery, AF attributable to reversible causes, a left ventricular ejection fraction below 35%, and New York Heart Association functional class III/IV congestive heart failure. A comprehensive list of all inclusion and exclusion criteria is provided in Supplemental Table 1.

### Randomization

After 1:1 randomization, patients undergo PVI with either the FARAPULSE PFA catheter or the Arctic Front cryoballoon. To conceal treatment assignment, a restricted randomization method employing permuted blocks with randomly varying block sizes of 2, 4, or 6 is employed. Additionally, study centers stratify the randomization process. The allocation sequence is based on computer-generated random numbers and integrated into the database.

### Preprocedural examinations, sedation, and LA access

Prior continuous anticoagulation with vitamin K antagonists or direct oral anticoagulants for ≥4 weeks or exclusion of LA thrombus ≤48 hours before ablation via transesophageal echocardiography and/or computed tomography is mandatory. Deep conscious sedation guided by a physician-led, nurse-administered protocol using midazolam, fentanyl, and propofol is used.^25^ Patients at high risk of sedation complications undergo general anaesthesia. LA access is achieved either through fluoroscopy- or transesophageal echocardiography–guided transseptal puncture using a standard transseptal sheath or by puncturing via the FARADRIVE sheath (Boston Scientific) as described previously in our institution.^18^^,^^26^ Heparin is administered to maintain an activated clotting time ≥350 seconds during the procedure.

### PFA procedure

The FARAPULSE PFA system consists of (1) a generator (FARASTAR; Boston Scientific), which produces short, high-electric-field pulses; (2) a 12F over-the-wire multipolar ablation catheter with either a 31 mm or 35 mm diameter (FARAWAVE; Boston Scientific); and (3) a 13F steerable sheath (FARADRIVE). The ablation catheter design consists of 5 splines, each containing 4 electrodes, totaling 20 ablation electrodes. These splines are connected at both their proximal and distal ends and can assume various shapes, ranging from a spherical “basket,” targeting the PV-to-LA conduction inside the PV to a fully deployed flat “flower” configuration, targeting the ostial PV-to-LA conduction. The application of the ablative electric field occurs in a bipolar manner between the catheter electrodes, and the pulse sequence is biphasic, activating all 20 electrodes simultaneously. As a result, a complex electrical field is generated around the catheter, leading to the permeabilization and destruction of vulnerable cells in close proximity.

After transseptal puncture, 0.5 mg of atropine is given to blunt vagal reactions potentially induced by the PFA applications. A J-shaped guidewire is used to cannulate the veins and the device is deployed inside the LA. PVI is performed with 4 PFA applications in basket configuration and 4 PFA applications in flower configuration per vein. Between pairs of PFA applications, the catheter is rotated by 30° to 40° after the first 2 applications in each configuration to cover the entire circumference. Ablation is performed using 2 kV pulses. PV isolation is verified at the end of the procedure using the FARAWAVE catheter in basket configuration in all PVs assessing entrance and exit block. In cases of residual PV conduction, additional PFA applications are delivered until PV isolation is achieved. No additional LA lesions outside the PV are permitted and no focal ablation catheters are used for completion of PVI.

Further specifications of the FARAPULSE PFA catheter in contrast to the Arctic Front cryoballoon are shown in Table 1.

**Table 1:** Comparison of the Arctic Front cryoballoon and the FARAPULSE Pulsed Field Ablation System

Ablation device	Arctic Front	FARAWAVE
Image of ablation catheter		
Ablation energy	Cryothermal	Pulsed electric field
Energy delivery	Whole balloon	All 20 electrodes
Deployed size	Standard (28 mm) Small (23 mm)	Standard (31 mm) Large (35 mm)
EGM recording electrodes	8 wired electrodes of 1 mm size 4 mm spacing (15 mm loop) 6 mm spacing (20 mm loop)	5 wired electrodes of 2 mm size (black circles) 16 mm spacing (standard) 20 mm spacing (large)
Typical duration of a single application	3–4 min	3 s
Delivery sheath	12F	13F
Patients treated worldwide so far	>1,000,000 patients in more than 80 countries	>50,000 patients
CE mark	August 2012	February 2021
FDA approval	August 2012	January 2024

EGM = electrogram; FDA = Food and Drug Administration.

### Cryoballoon ablation procedure

Cryoablation is performed with the Arctic Front cryoballoon. The Arctic Front cryoballoon is 28 mm or 23 mm in diameter, uses a 20 mm circular mapping catheter (Achieve Advance; Medtronic), and a steerable sheath (15F FlexCath Advance; Medtronic). The minimal target temperature to achieve an effective cryoapplication is below –40 °C.

After successful LA access, the standard transseptal sheath is replaced by the corresponding steerable sheath (FlexCath). Following introduction, the cryoballoon is placed at the ostium of each PV to occlude the veins (verified by dye injection). In case of an effective freeze (disappearance of all local PV signals or reaching a temperature of –40 °C before 60 seconds if local signals are absent), cryoablation is continued for 2 additional minutes after effect (“time-to-effect plus 2 minutes strategy”).^27^ In case of an ineffective freeze not resulting in PV isolation or not achieving minimal target temperatures in the absence of local signals, the balloon and/or guidewire are repositioned, aiming for better occlusion of the PV, and a new lesion will be delivered. No additional LA lesions outside the PV are permitted and no focal ablation catheters are used for completion of PVI. For patients confirmed to have typical right atrial flutter, the option for cavotricuspid isthmus ablation using radiofrequency ablation is available at the discretion of the operators. The procedural endpoint will be PV isolation and are assessed at the end of the procedure with the circular mapping catheter (Achieve; Medtronic) for all PVs without a waiting period. In case of recurrence, additional freezes are allowed.

### 3-dimensional LA voltage map for lesion assessment

The first 25 patients in each intervention group will undergo postablation electroanatomic mapping (EAM) of the LA using a 3 dimensional EAM system (RHYTHMIA; Boston Scientific) to quantify the proportion of isolated PVs, the proportion of isolated carinas and the lesion size. A dedicated high-resolution mapping catheter (Orion; Boston Scientific) will be introduced to create a high-density voltage map of the LA using the FlexCath or FARADRIVE sheath already present.

Given that the workflow tested in our study is PFA/cryoballoon procedures guided by fluoroscopy only without the use of a 3-dimensional (3D) mapping system, the acute endpoint of the procedure will be assessed before the beginning of the 3D mapping part. In case a reconnected PV is observed during 3D-EAM, no additional ablation is allowed because it would bias the overall study.

### Implantable cardiac monitor

To ensure continuous rhythm monitoring during follow-up, an ICM (Reveal LinQ; Medtronic) with remote monitoring capabilities is implanted in all patients of both groups upon completion of the ablation procedure. Procedural sedation is maintained throughout the implantation procedure, and local anesthesia is administered to the precordial implantation site for patient comfort. The ICM incorporates an AF detection algorithm that continuously analyzes beat-to-beat variability of cardiac cycles on a 2-minute electrocardiogram strip. This allows for precise determination of arrhythmia recurrence timing and accurate quantification of AF burden (hours in AF per day and percentage of overall time in AF). The ICM parameters programmed for this study are outlined in the Supplemental Table 2. ICM data will be uploaded to a dedicated Medtronic CareLink research account with only coded data transmitted. The transmitted ICM data will be analyzed centrally in the ICM Core Laboratory at the Inselspital – University Hospital Bern, Switzerland.

### Postablation follow-up

Blood sampling for postablation high-sensitive troponin release is obtained in the morning of the first postablation day.

Antiarrhythmic drugs are allowed during the first 3 months (blanking period) but will be discontinued after 3 months by the latest. Following discharge, daily automatic ICM data transmissions will be used to assess and record atrial tachyarrhythmia events and burden. Additionally, manual transmissions are performed once weekly. Clinical follow-up visits occur at 3, 6, 12, 24, and 36 months at the study center enrolling the patient or by external cardiologists. Telephone follow-ups are conducted at 3 months ± 2 weeks, 12 ± 2 months, 24 ± 2 months, and 36 ± 2 months to assess hospital or emergency room admissions, electrical cardioversions, repeat ablation procedures, stroke/transient ischemic attack, or death. During the 3-month telephone follow-up, a final evaluation of delayed procedural complications contributing to the safety endpoint is conducted. The 36-month telephone follow-up is the last follow-up. Quality-of-life (QoL) questionnaires are sent to patients by mail after 3 and 12 months to monitor QoL progression postablation by the EQ-5D-5L questionnaire.^28^ Refer to Table 2 for a summary of the enrollment, interventions, and assessments schedule.

**Table 2:** Schedule of enrollment, interventions, and assessments

	Enrollment	Procedure		Follow-up			
	≥1 d prior to ablation	0	Hospital DC	3 mo	12 mo	24 mo	36 mo
Eligibility screen	X						
Informed consent	X						
Clinical examination	(X)		(X)				
Laboratory investigation	(X)		(X)				
12-lead ECG	(X)		(X)				
Echocardiography	(X)						
QoL questionnaire	X			X	X		
Cardiac CT or MRI	(X)						
PV isolation		(X)					
3D-EAM (substudy)		X∗					
ICM implantation		X					
ICM data transmission							
Telephone call				X	X	X	X
Safety endpoint assessment			X	X			
Primary endpoint assessment					X		

In parentheses are routine assessments and interventions (not study specific).

3D = 3-dimensional; CT = computed tomography; DC = discharge; EAM = electroanatomic mapping; ECG = electrocardiogram; ICM = implantable cardiac monitor; MRI = magnetic resonance imaging; PV = pulmonary vein; QoL = quality of life.

∗ Performed only in the first 25 patients in each intervention group.

## Study outcomes

### Primary endpoint

The primary endpoint is time to first recurrence of any atrial tachyarrhythmia (AF and/or organized atrial tachyarrhythmia) between days 91 and 365 following ablation procedure, as detected on continuous ICM. Atrial tachyarrhythmia recurrence is defined as continuous arryhthmia duration lasting 30 seconds or longer on a detected ICM episode of minimally 120 seconds (the minimum programmable episode interval). The 3-month blanking period adheres to the current recommendations for defining outcomes in AF ablation trials.^29^

Regarding the assessment of the primary endpoint, every possible atrial tachyarrhythmia episode of minimally 120 seconds duration and transmitted by the patient’s ICM is collected in a chronological order. These potential arrhythmia events are then independently reviewed and adjudicated by an independent clinical events committee comprised of 3 experienced electrophysiologists blinded to treatment allocation.

### Secondary endpoints

The procedural safety endpoint is a composite of (1) cardiac tamponade requiring pericardiocentesis, (2) persistent phrenic nerve palsy lasting >24 hours, (3) serious vascular complications requiring intervention, (4) stroke/transient ischemic attack, (5) atrioesophageal fistula, or (6) death. The safety endpoint is assessed during catheter ablation, the time until discharge, and the following 30 days. Information on safety events are collected throughout the trial by the investigators or their designees. All primary safety events will be reviewed and adjudicated by the clinical events committee members.

Other key secondary endpoints include (1) procedure duration, LA indwelling, and fluoroscopy times; (2) proportion of isolated PVs and carinas between the PVs in postablation 3D-EAM (only the first 25 patients in each study group); (3) AF burden; (4) proportion of patients with recurrence of any atrial tachyarrhythmia between days 0 and 90 postablation (blanking period); (5) proportion of patients undergoing a repeat ablation; (6) QoL changes at 3 and 12 months compared with baseline; and (7) modified analysis of the primary endpoint using a blanking period duration of 2 months as proposed in the recently renewed AF ablation consensus document.^30^ All secondary procedural and follow-up endpoints are listed in the Supplemental Table 3.

### Sample size

Using continuous ICM data, the occurrence of the primary endpoint is expected at 40% based on the data from the recent Cryoballoon or Radiofrequency Ablation for Atrial Fibrillation Assessed by Continuous Monitoring: A Randomized Clinical Trial study,^31^ and the Cryoballoon or Radiofrequency Ablation for Paroxysmal Atrial Fibrillation trail.^32^ With a sample size of 99 per group and a 1-sided 0.025 significance level, the study will have 80% power (using log-rank test) with the noninferiority margin set to 20% comparing the 2 single-shot ablation technologies. This noninferiority margin and power is identical to the noninferiority margin in the Cryoballoon or Radiofrequency Ablation for Atrial Fibrillation Assessed by Continuous Monitoring: A Randomized Clinical Trial study, which is one of the most highly cited articles on AF ablation in recent years and may be considered relevant and representative of our field.^31^ The upper limit of the 2-sided 95% confidence interval for the difference in the cumulative incidence comparing the FARAPULSE PFA catheter vs the Arctic Front cryoballoon had to exclude 1.2 times the percentage points of the cumulative incidence in the control device (Artic Front) for the primary outcome to define noninferiority of the experimental device (FARAPULSE). In accordance with the Food and Drug Administration regulations, the noninferiority hypothesis was tested using a 1-sided alpha of 0.025, which corresponds to a 2-sided alpha of 0.05.

Factoring in a 5% loss to follow-up, 105 patients per group will be randomized, resulting in a total study population of 210 (Supplemental Table 4).

### Data management

Study data will be collected and managed using REDCap (Research Electronic Data Capture) electronic data capture tools hosted at the Clinical Trials Unit (CTU) at the University Hospital of Bern.^33^^,^^34^ REDCap is a secure, Web-based software platform designed to support data capture for research studies, providing (1) an intuitive interface for validated data capture; (2) audit trails for tracking data manipulation and export procedures; (3) automated export procedures for seamless data downloads to common statistical packages; and (4) procedures for data integration and interoperability with external sources. The case report forms are implemented electronically. All electronic data are encrypted, password protected and stored on a secure network at the CTU of the University of Bern. Regular evaluations of data integration and quality, management and resolution of data discrepancies, tracking of adverse event information and database quality control will be performed. At the conclusion of the study, the CTU Bern will lock the clinical data and perform the final analysis of the trial results.

### Statistical analysis

A statistician, who is blinded to treatment allocation, will perform analyses. The principal investigators will have full access to the data and will vouch for the data and the analysis. Analyses are conducted according to the intention-to-treat principle meaning that patients are analyzed based on the treatment arm to which they were originally allocated. The primary analysis is in the per-protocol population with confirmatory testing in all the randomized patients, in both these populations using the intention-to-treat principle. Hence, noninferiority will only be claimed for the FARAPULSE PFA catheter if the analysis in both populations show that the upper limit of the confidence interval of the rate ratio using Mantel-Cox time to first recurrence of any atrial tachyarrhythmia between days 91 and 365 postablation comparing FARAPULSE vs Arctic Front does not cross 20% (rate ratio of 1.2), using a 1-sided test with alpha of 5%.

The per-protocol population is defined as all patients in which (1) the randomized device was used to initiate the ablation (irrespective of whether this ablation was completed successfully, or whether a second device was used to complete the ablation) and (2) none of the inclusion and exclusion criteria were breached.

Primary analysis will take place after all patients have completed 1-year follow-up. For the primary analyses (FARAPULSE vs Arctic Front), unadjusted survival curves are estimated by the Kaplan-Meier method and compared by log rank tests. Unadjusted rate ratios and confidence intervals for rate ratios are derived from Mantel-Cox models. The proportional hazards assumption is assessed by visual inspection of the log-negative-log plot and through a formal test of the interaction term group × time at an alpha of 0.05. Secondary endpoints expressed as time to event are analyzed similarly using Kaplan-Meier survival curves and a log-rank test. All tests are conducted at an alpha level of 0.05. Basic assumptions are verified prior to analysis. In case of noninferiority for the primary endpoint, a superiority analysis is performed using a 2-tailed significance alpha level of 0.05.

Comparisons between the 2 treatment groups are performed using demographic, clinical, procedural, and imaging characteristics, and tested if postrandomization. Continuous variables are presented as mean ± SD or median and interquartile range depending on their distribution. Categorical variables are stated as number and proportion. Characteristics are compared between the 2 treatment groups by means of unpaired *t* tests for continuous variables and chi-square tests or Fisher’s exact tests for categorical variables and reported *P* values will be 2-sided. If a normal distribution cannot be assumed, nonparametric tests are considered to compare patient characteristics.

### Funding and Sponsorship

This investigator-initiated study is funded by institutional research grants from Inselspital – University Hospital Bern and the 10.13039/100016015University Hospital Basel and a research grant from Boston Scientific. Boston Scientific is cofinancing the project with a total of 393,450 CHF. The study is sponsored by Insel Gruppe AG, Department of Cardiology, Inselspital – Bern University Hospital, 10.13039/100009068University of Bern.

### Ethical considerations

The local ethical committees (2022-D0024) have approved this study. The trial will be conducted in accordance with the principles of Good Clinical Practice and the Declaration of Helsinki.

Participation in the study assumes that patients have already committed to undergoing catheter ablation for paroxysmal AF. The catheter ablation procedure employed in this trial mirrors the established treatment approach for AF and is not experimental. Consequently, the risks associated with participation are equivalent to those of conventional AF ablation and remain unaffected by trial enrollment.

## Discussion

The SINGLE SHOT CHAMPION study is the first randomized controlled trial with blinded ICM based endpoint adjudication to compare the novel PFA modality (FARAPULSE) with the currently well-established cryoballoon ablation (Arctic Front) for PVI in patients with paroxysmal AF.

Single-shot approaches for PVI offer multiple advantages compared with point-by-point radiofrequency ablation including shorter learning curve and procedure times.^31^ The Arctic Front cryoballoon is now the most frequent used single-shot device. However, procedure-related complications associated to nonselective, thermal energy ablation such as atrioesophageal fistula and persisting phrenic nerve palsy occur.^9^ The novel FARAPULSE PFA system delivering high-voltage electrical fields potentially minimizes these risks by selective myocardial tissue ablation. Nevertheless, the first large postmarket registry (n = 1817 patients) reported occurrence of transient (n = 6) and even persistent (n = 1) phrenic nerve palsy after PVI with the FARAPULSE PFA system but no atrioesophageal fistula.^17^

The recently published, first randomized controlled ADVENT study (Pulsed Field or Conventional Thermal Ablation for Paroxysmal Atrial Fibrillation) comparing PFA and thermal ablation (cryoballoon and radiofrequency ablation) for PVI showed similar efficacy between both groups.^20^ One main limitation of this study may be the lack of continuous rhythm monitoring, possibly leading to underestimation of recurrence rates and AF burden. SINGLE SHOT CHAMPION addresses this limitation by providing continuous rhythm monitoring during follow-up via ICM in all participants. As shown previously, the choice of an adequate monitoring strategy is crucial for a reliable comparison of long-term efficacy between ablation technologies.^24^

## Conclusion

The SINGLE SHOT CHAMPION study is the first randomized controlled, multicenter study comparing the safety and long-term efficacy of the novel FARAPULSE PFA catheter with the standard-of-practice Arctic Front cryoballoon using ICM based follow-up data. It will contribute to a deeper understanding of the clinical performance and safety of the 2 systems and inform future selection of the optimal catheter ablation system in AF patients.

## Appendix Supplementary Data

Single Shot Champion
