# Corrigendum to “Comparison of Cryoballoon vs. Pulsed Field Ablation in Patients with Symptomatic Paroxysmal Atrial Fibrillation (SINGLE SHOT CHAMPION): Study protocol for a randomized controlled trial”, *Heart Rhythm O^2^,* Volume 5, Issue 7, (July 2024) P460-467

**DOI:** 10.1016/j.hroo.2025.03.002

**Published:** 2025-04-07

The authors of the Design Paper by Maurfhofer et al., which published in the July 2024 issue of *Heart Rhythm O*^*2*^ (2024;5:460-7, DOI: 10.1016/j.hroo.2024.05.008), have identified three important errors that contradict the intended trial design and the protocol approved by the Research Ethics Committee in 2022:

• The trial is a non-inferiority trial with an absolute margin of 20% for the difference in cumulative incidence. The article erroneously describes the margin as relative rather than absolute. The sample size calculation (99 patients per group) was based on an absolute margin of 20% for the difference in cumulative incidence, not a relative margin of 20% for the rate ratio. If the margin were relative, the required sample size per group would have been 589, not 99.
• Despite the non-inferiority design, the log-rank test was mentioned multiple times. However, this test is not suitable for non-inferiority analyses.
• The article mentions using a Mantel-Cox model to test non-inferiority. However, Mantel-Cox rate ratios are appropriate for a non-inferiority design with a relative margin but not for one with an absolute margin. Instead, the difference in cumulative incidence should be used, as this aligns with the trial’s absolute margin.

The first paragraph in the “Sample size” section on page 465 should read as follows:

Using continuous ICM data, the occurrence of the primary endpoint is expected at 40% based on the data from the recent Cryoballoon or Radiofrequency Ablation for Atrial Fibrillation Assessed by Continuous Monitoring: A Randomized Clinical Trial study,^31^ and the Cryoballoon or Radiofrequency Ablation for Paroxysmal Atrial Fibrillation trial.^32^ With a sample size of 99 per group and a 1-sided 0.025 significance level, the study will have 80% power with the noninferiority margin set to 20% comparing the 2 single-shot ablation technologies. This noninferiority margin and power is identical to the noninferiority margin in the Cryoballoon or Radiofrequency Ablation for Atrial Fibrillation Assessed by Continuous Monitoring: A Randomized Clinical Trial study, which is one of the most highly cited articles on AF ablation in recent years and may be considered relevant and representative of our field.^31^ The upper limit of the 2-sided 95% confidence interval for the difference in the cumulative incidence comparing the FARAPULSE PFA catheter vs the Arctic Front cryoballoon had to exclude 20 percentage points for the primary outcome to define noninferiority of the experimental device (FARAPULSE). In accordance with the Food and Drug Administration regulations, the noninferiority hypothesis was tested using a 1-sided alpha of 0.025, which corresponds to a 2-sided alpha of 0.05.

The first three paragraphs of the “Statistical analysis” section on page 465 should read as follows:

A statistician, who is blinded to treatment allocation, will perform analyses. The principal investigators will have full access to the data and will vouch for the data and the analysis. Analyses are conducted according to the intention-to-treat principle meaning that patients are analyzed based on the treatment arm to which they were originally allocated. The primary analysis is in the per-protocol population with confirmatory testing in all the randomized patients, in both these populations using the intention-to-treat principle. Hence, noninferiority will only be claimed for the FARAPULSE PFA catheter if the analysis in both populations show that the upper limit of the confidence interval of the difference in cumulative incidence of the first recurrence of any atrial tachyarrhythmia between days 91 and 365 postablation comparing FARAPULSE vs Arctic Front does not cross 20%, using a 1-sided test with alpha of 2.5%.

The per-protocol population is defined as all patients in which (1) the randomized device was used to initiate the ablation (irrespective of whether this ablation was completed successfully, or whether a second device was used to complete the ablation) and (2) none of the inclusion and exclusion criteria were breached.

Primary analysis will take place after all patients have completed 1-year follow-up. For the primary analyses (FARAPULSE vs Arctic Front), unadjusted survival curves are estimated by the Kaplan-Meier method and compared. Unadjusted rate ratios and confidence intervals for rate ratios are derived from Cox models. The proportional hazards assumption is assessed by visual inspection of the log-negative-log plot and through a formal test of the interaction term group X time at an alpha of 0.05. Secondary endpoints expressed as time to event are analyzed similarly using Kaplan-Meier survival curves. All tests are conducted at a two-sided alpha level of 0.05. Basic assumptions are verified prior to analysis. In case of noninferiority for the primary endpoint, a superiority analysis is performed using a 2-tailed significance alpha level of 0.05.

The authors regret these errors and would like to apologize for any inconvenience caused.

