# Supplementary material for: Comparison of Cryoballoon vs. Pulsed Field Ablation in Patients with Symptomatic Paroxysmal Atrial Fibrillation (SINGLE SHOT CHAMPION): Study protocol for a randomized controlled trial
Source: Heart Rhythm O2. 2024 Jun 3;5(7):460–7. doi: 10.1016/j.hroo.2024.05.008 (PMC11305878; doi:10.1016/j.hroo.2024.05.008)
Supplement: Single Shot Champion [file mmc1.docx]

# **Supplementary Material**

| **Table S1** Inclusion and exclusion criteria |
| --- |
| **Inclusion criteria:** |
| - Paroxysmal atrial fibrillation documented on a 12 lead ECG or Holter monitor (lasting ≥30 seconds) within the last 24 months. According to current guidelines, paroxysmal is defined as any AF that converts to sinus rhythm within 7 days either spontaneously or by pharmacological or electrical cardioversion |
| - Candidate for ablation based on current AF guidelines |
| - Continuous anticoagulation with Vitamin-K-Antagonists or a NOAC for ≥4 weeks prior to the ablation; or a TEE and/or CT that excludes LA thrombus ≤48 hours before ablation |
| - Age of 18 years or older on the date of consent |
| - Informed consent as documented by signature |
|  |
| **Exclusion criteria:** |
| - Previous LA ablation or LA surgery |
| - Persistent AF |
| - AF due to reversible causes (e.g. hyperthyroidism, cardiothoracic surgery) |
| - Intracardiac thrombus |
| - Pre-existing PV stenosis or PV stent |
| - Pre-existing hemidiaphragmatic paralysis |
| - Contraindication to anticoagulation or radiocontrast materials |
| - Prior mitral valve surgery |
| - Severe mitral regurgitation or moderate/severe mitral stenosis |
| - Myocardial infarction during the 3-month period preceding the consent date |
| - Ongoing triple therapy |
| - Cardiac surgery during the three-month interval preceding the consent date or scheduled cardiac surgery/TAVI procedure |
| - Significant congenital heart defect (including atrial septal defects or PV abnormalities but not including PFO) |
| - NYHA class III or IV congestive heart failure |
| - LVEF <35% |
| - Hypertrophic cardiomyopathy (wall thickness >1.5 cm) |
| - Significant chronic kidney disease (eGFR <30 ml/min) |
| - Uncontrolled hyperthyroidism |
| - Cerebral ischemic event (stroke or TIA) during the six-month interval preceding the consent date |
| - Ongoing systemic infections |
| - History of cryoglobulinemia |
| - Cardiac amyloidosis |
| - Pregnancy^*^ |
| - Life expectancy less than one year per physician opinion |
| - Currently participating in any other clinical trial, which may confound the results of this study. |
| - Unwilling or unable to comply fully with study procedures and follow-up. |
| * To exclude pregnancy a blood test (HCG) is used in women < 50 years.  **Abbreviations:** AF = atrial fibrillation; CT = computer tomography; ECG = electrocardiogram; eGFR = estimated glomerular filtration rate; HCG = human chorionic gonadotropin; LA = left atrial; LVEF = left ventricular ejection fraction; NOAC = novel oral anticoagulants; NYHA = New York Heart Association; PV = pulmonary vein; TAVI = transcatheter aortic valve implantation;TEE = transesophageal echocardiography; TIA = transient ischemic attack. |

## **Table S1**

| **Table S2** Arrhythmia detection algorithm for implantable cardiac monitoring | |
| --- | --- |
| **Parameters** | **Settings** |
| Tachycardia | ON / 182 bpm / 48 beats |
| Bradycardia | ON / 30 bpm / 8 beats |
| Pause | ON / 4.5 sec |
| Symptom | OFF |
| AT/AF | AF only |
| AT/AF detection | AF only |
| AF detection sensitivity | Balanced sensitivity |
| Ectopy rejection | Nominal |
| AT/AF recording threshold | All episodes |
| **Abbreviations:** AF = atrial fibrillation; AT = atrial tachycardia; bpm = beats per minute. | |

## **Table S2**

## **Table S3**

| **Table S3 Secondary procedural and follow-up endpoints.** | |
| --- | --- |
| **1) Safety Endpoint:** | |
| Composite of (i) cardiac tamponade requiring drainage, (ii) persistent phrenic nerve palsy lasting > 24 hours, (iii) serious vascular complications requiring intervention, (iv) stroke/TIA, (v) atrioesophageal fistula, or (vi) death. | |
| **2) Secondary Procedural Endpoints:** | |
| - Total procedure time | - Post-ablation 3D electro-anatomical mapping (first 25 patients in each study group): |
| - Total LA indwelling time | - Proportion of isolated pulmonary veins |
| - Total fluoroscopy time | - Proportion of isolated carinas |
| - Total radiation dose | - Lesion size |
| - Contrast agent usage (ml) | - Clinically manifest vasospasm during procedure |
| - Increase in hsTroponin on day 1 post-ablation |  |
| **3) Secondary Endpoints during Follow-Up:** | |
| - Time to first recurrence of atrial tachyarrhythmia between days 1 and 90 after ablation. | |
| - Time to first recurrence of atrial tachyarrhythmia between days 61 and 365 after ablation (adjusted blanking period duration of 2 months) | |
| - Arrhythmia burden between days 0-90 evaluated based on continuous ICM | |
| - Arrhythmia burden between days 91 and 365 evaluated based on continuous ICM | |
| - Arrhythmia burden between days 365 until explantation/or end of life of the ICM | |
| - Comparison of the prevalence of the type of arrhythmia recurrences during follow-up being AF or organized atrial arrhythmias (AFL or AT) | |
| - Average heart rates in ICM documentation in months 1, 2 and 3 after ablation procedure | |
| - Proportion of patients admitted to the hospital or emergency room because of documented recurrence of atrial arrhythmias | |
| - Proportion of patients undergoing electrical cardioversion because of documented recurrence of atrial arrhythmias | |
| - Proportion of patients undergoing a repeat ablation procedure because of documented recurrence of atrial arrhythmias | |
| - Re-initiation of AAD during follow-up | |
| - Number of reconnected pulmonary veins, sites of reconnection and size of antral scar area evaluated during redo procedures at one of the study centres | |
| - Evolution of QoL after 3 and 12 months | |
| - Proportion of stroke/TIA after 3, 12, 24 and 36 months | |
| - Proportion of death, cardiovascular or non-cardiovascular after 3, 12, 24 and 36 months | |
| **Abbreviations:** AAD = antiarrhythmic drug; AF = atrial fibrillation; AFL = atrial flutter; AT = atrial tachycardia; ICM = implantable cardiac monitor; LA = left atrial; QoL = quality of life; TIA = transient ischemic attack. | |

| **Table S4** Assumptions regarding the sample size calculations in similar trials in relation to our study. | | | | | |
| --- | --- | --- | --- | --- | --- |
|  | Estimated Event Rate | Non-inferiority margin | Ratio | Observed Event Rate | Type of follow-up |
| FIRE & ICE (Kuck KH, NEJM, 2016) | 30% | 10% | 0.33 | 35% | Holter ECG |
| CIRCADOSE (Andrade JG, Circulation, 2019) | 35% | 20% | 0.57 | 48% | ICM |
| SINGLE SHOT CHAMPION | 40% | 20% | 0.50 | n.a. | ICM |
| **Abbreviations:** ECG = electrocardiography; ICM = implantable cardiac monitor. | | | | | |

## **Table S4**
